# Efficacy and safety of FOLFIRINOX as salvage treatment in advanced biliary tract cancer: an open-label, single arm, phase 2 trial

**DOI:** 10.1038/s41416-019-0698-9

**Published:** 2020-01-10

**Authors:** Ali Belkouz, Judith de Vos-Geelen, Ron A. A. Mathôt, Ferry A. L. M. Eskens, Thomas M. van Gulik, Martijn G. H. van Oijen, Cornelis J. A. Punt, Johanna W. Wilmink, Heinz-Josef Klümpen

**Affiliations:** 1 Amsterdam UMC, Department of Medical Oncology, University of Amsterdam, Cancer Center Amsterdam, Meibergdreef 9, 1105 AZ Amsterdam, The Netherlands; 2 Maastricht UMC+, GROW - School for Oncology and Developmental Biology, Department Of Internal Medicine Division of Medical, P. Debyelaan 25, 6229 HX Maastricht, The Netherlands; 3 Amsterdam UMC, Department of Pharmacy, University of Amsterdam, Cancer Center Amsterdam, Meibergdreef 9, 1105 AZ Amsterdam, The Netherlands; 4 Erasmus MC, Department of Medical Oncology, Doctor Molewaterplein 40, 3015 GD Rotterdam, The Netherlands; 5 Amsterdam UMC, Department of Surgery, University of Amsterdam, Cancer Center Amsterdam, Meibergdreef 9, 1105 AZ Amsterdam, The Netherlands

**Keywords:** Biliary tract cancer, Chemotherapy

## Abstract

**Background:**

No standard treatment is available for advanced biliary tract cancer (BTC) after first-line therapy with gemcitabine plus cisplatin (GEMCIS). The objective of this study was to evaluate safety and anti-tumour activity of fluorouracil, leucovorin, irinotecan plus oxaliplatin (FOLFIRINOX) as salvage treatment in patients with previously treated advanced BTC.

**Methods:**

In this two-stage phase 2 study, patients with advanced BTC who had disease progression or unacceptable toxicity after ≥3 cycles of GEMCIS were eligible. Primary endpoints were safety and efficacy (defined as objective response rate, ORR). In stage one, ten patients were treated with FOLFIRINOX every 2 weeks. In stage two, an additional 20 patients were enrolled at a starting dose as defined in stage one, provided that in stage ≥1 objective response or ≥2 stable diseases were observed and ≤3 patients had serious adverse events (SAEs) within the first 6 weeks of treatment. Secondary endpoints were progression-free survival (PFS) and overall survival (OS).

**Results:**

Forty patients were screened for eligibility and 30 patients were enrolled. In stage one, one patient had a partial response and five patients had stable disease. One patient had a SAE during the first 6 weeks of treatment, and five patients required a dose reduction due to adverse events. The most common grade 3–4 adverse events in stage one were neutropaenia, mucositis and diarrhoea. Stage two was initiated with FOLFIRINOX in an adapted dose. In stage two, grade 3–4 neutropaenia, diarrhoea, nausea and vomiting were the most common adverse events. The ORR, median PFS and OS in all patients were 10%, 6.2 and 10.7 months, respectively.

**Conclusions:**

In patients with advanced BTC who progressed after or were intolerant to GEMCIS, FOLFIRINOX can be administered safely and could be considered as an option for salvage treatment in these patients.

**Clinical trial registration:**

ClinicalTrials.gov Identifier NCT02456714.

## Background

Biliary tract cancer (BTC) is a malignancy of intrahepatic and extrahepatic bile ducts including the gallbladder. It has a low incidence in Europe and North America, but it is more common in east-Asian and south American countries.^1^ Systemic chemotherapy has shown to improve survival and quality of life in patients with advanced BTC, with gemcitabine plus cisplatin (GEMCIS) currently being considered as standard of care.^2,3^ After disease progression or unacceptable adverse events of first-line chemotherapy, no standard second-line treatment is available. Preliminary data from the phase 3 ABC-06 trial suggest that modified fluorouracil/leucovorin and oxaliplatin (FOLFOX) show significant but very limited benefit compared to active symptom control (ASC) in second-line setting.^4^ Novel and more effective treatment options are therefore warranted.

In clinical practice, the percentage of patients receiving second-line systemic treatment varies greatly, and this treatment usually is fluoropyrimidine-based.^5,6^ Lamarca et al.^7^ have performed a meta-analysis of 25 studies with a total of 761 patients treated with second-line chemotherapy, mostly gemcitabine and/or fluorouracil-based. Efficacy was modest with a progression-free survival (PFS) of 3.2 months and a median overall survival (OS) of 7.2 months, respectively.^7^

Second-line fluoropyrimidine monotherapy has shown modest activity in BTC with a median PFS of 2.5–5.5 months and a median OS of 7.5–13.5 months, respectively,^8–10^ whereas fluoropyrimidine-based combination therapy with either oxaliplatin (FOLFOX) or irinotecan (FOLFIRI) showed a median PFS of 1.6–3.9 months and a median OS of 4.4–8.4 months.^11–13^

The combination fluorouracil, leucovorin, irinotecan plus oxaliplatin (FOLFIRINOX) has only been given anecdotally as second-line treatment in BTC,^14–18^ but has shown encouraging results in advanced pancreatic cancer.^19^ In order to assess the potential activity of this regimen as a salvage treatment, we conducted a phase 2 study to determine efficacy and safety of this regimen in patients with advanced BTC previously treated with GEMCIS.

## Methods

### Study design and participants

This trial was performed according to the Bryant and Day two-stage design^20^ to enrol a total of 30 patients with advanced BTC previously treated with GEMCIS. In the first stage, ten patients were treated with standard doses of FOLFIRINOX (oxaliplatin 85 mg/m^2^, irinotecan 180 mg/m^2^, leucovorin 400 mg/m^2^ and fluorouracil bolus 400 mg/m^2^, followed by continuous infusion of 2400 mg/m^2^ fluorouracil over 46 h). Subsequently, stage two of this study was initiated if at least one objective response rate (ORR) or two stable diseases had been observed and not more than three patients had experienced treatment-related serious adverse events (SAEs) within the first 6 weeks of treatment.

Patients aged ≥18 years with Eastern Cooperative Group performance status (ECOG PS) of ≤ 1 and histologically or cytologically confirmed advanced cholangiocarcinoma or gallbladder carcinoma with disease progression during or after ≥ 3 cycles of GEMCIS or intolerance to this treatment toxicities were enrolled in this trial. Other inclusion criteria included measurable disease according to RECIST1.1, and adequate bone-marrow, liver and kidney function. Patients with combined hepatocellular-cholangiocarcinoma were excluded.

All patients provided written informed consent. The institutional review boards and Medical ethics committee at the Amsterdam UMC reviewed and approved the protocol. The study was performed in accordance with the Declaration of Helsinki and the Good Clinical Practice guidelines. The study was registered at Clinical Trials.gov (NCT02456714) on 28 May 2015.

### Procedures

#### Treatment

In stage one of the study, patients received a starting dose of oxaliplatin (85 mg/m^2^), leucovorin (400 mg/m^2^), irinotecan (180 mg/m^2^) and fluorouracil bolus (400 mg/m^2^) followed by continuous infusion of fluorouracil (2400 mg/m^2^) over 46 h, every 14 days for a maximum of 12 cycles. Toxicities in stages one and two were managed by dose reduction or interruption depending on the most likely cause of toxicity and according to a predefined schedule. Stage two was initiated if no more than three patients in stage one had a SAE, within the first 6 weeks of treatment and at least one patient with an objective response or two patients with stable disease were observed in stage one. If more than four patients in stage one required any dose reduction due to treatment-related adverse events, a standard dose reduction defined in stage one would be initiated as a standard starting dose in stage two. The use of therapeutic or prophylactic granulocyte colony stimulating factor (G-CSF) was not allowed according to the study protocol.

#### Assessments

At baseline evaluation and at start of every cycle, patients were assessed by physical examination, ECOG PS and laboratory exams. Serum carbohydrate antigen 19–9 (CA 19-9) was measured at baseline and every four cycles. Computed tomographic scans of the chest, abdomen and pelvis were performed at baseline, and after every four cycles during the active treatment period followed by every 8 weeks after the treatment had been stopped. Disease assessment was done until disease progression or death. Tumour response was assessed according to RECIST1.1. Adverse events were graded using the National Cancer Institute Common Terminology Criteria (version 4.0).

### Outcomes

The primary endpoints were adverse events and the ORR. Secondary endpoints were PFS and OS. PFS and OS were defined as the time from the date of inclusion to the date of radiological or clinical disease progression and date of death from any cause, respectively. Insufficient quality of life data were collected for analysis because of a technical error.

### Statistical analysis

After inclusion of ten patients in stage one, we evaluated whether the FOLFIRINOX dosage was tolerable and could be used in stage two of this study. The ORR (partial plus complete response) was calculated for each stage separately as well as combined. In the final analyses, we combined patients from both stages. Patients receiving at least one dose of study medication were included in the final safety and efficacy analyses. The software R version 3.5.1 was used for statistical analysis. Baseline characteristics were summarised by descriptive statistics. Time-to-event variables were calculated using Kaplan–Meier method. Univariable Cox proportional hazard regression analysis was used to assess prognostic factors for PFS and OS.

## Results

Between 1 May 2016, and 22 March 2018, 40 patients were screened for participation and 30 patients were enrolled in this study. All patients had histologically or cytologically proven advanced BTC. Eleven patients (36.7%) had a perihilar cholangiocarcinoma, seven patients (23.3%) had a distal cholangiocarcinoma, five patients (16.7%) had an intrahepatic cholangiocarcinoma, six patients (20%) had a gallbladder carcinoma and one patient (3.3%) had an ampulla of Vater carcinoma (Table 1). Twenty-three patients (76.7%) had distant metastases at inclusion and seven patients (23.3%) had locally advanced disease. Patients had received previously a median number of six cycles (IQR 5–7.8) of GEMCIS. A total of 28 patients (93.3%) received a maximum of eight cycles of GEMCIS and the remaining two patients received more than eight cycles. The best response to GEMCIS was partial response in six patients (20.0%), stable disease in 14 patients (47.0%), disease progression in seven patients (23.0%) and unknown in three patients (10.0%). Twenty-nine (96.7%) patients had disease progression during the treatment with GEMCIS or follow-up, and one patient (3.3%) had intolerance for this regimen. The median time between last dose of GEMCIS and the start of (modified) FOLFIRINOX was 2.3 months (IQR 1.7–4.5).

**Table 1:** Baseline characteristics.

	Total (*N* = 30)	
	*N*	%
Median age, years (range)	60 (38–74)	
Sex		
Male	19	63.3
Female	11	36.7
ECOG performance status		
0	21	70
1	9	30
Primary site of disease		
Cholangiocarcinoma	23	76.7
Intrahepatic	5	16.7
Perihilar	11	36.7
Distal	7	23.3
Gallbladder	6	20
Ampulla of Vater	1	3.3
Extent of disease at study entry		
Metastatic	23	76.7
Locally advanced	7	23.3
Number of metastatic sites		
1 site	10	43.5
2 or more sites	13	56.5
Location of distant metastases		
Liver only^a^	7	30.4
Other distant locations	16	69.6
Previous treatments		
Curative-intent surgery	12	40.0
Only explorative laparotomy	5	16.7
Adjuvant chemotherapy	1	3.3
Gemcitabine plus cisplatin treatment	30	100
Median number of cycles (IQR)	6 (5–7.8)	
Best response		
Partial response	6	20
Stable disease	14	47
Progressive disease	7	23
Unknown	3	10
Time between last dose of GEMCIS and start of (modified) FOLFIRINOX, months (IQR)	2.3 (1.7–4.5)	
Median CA 19-9 concentration (IQR), U/mL	667 (97–4336)	
Median total bilirubin, μmol/L	7.0 (5.0–9.0)	
Subsequent treatment	9	30
Retreatment with modified FOLFIRINOX schedule	2	6.7
Trastuzumab plus pertuzumab	1	3.3
Tremelimumab	1	3.3
ATR inhibitor	1	3.3
CriPec^®^ nanoparticles with docetaxel	1	3.3
Metformine and chloroquine	1	3.3
Gemcitabine monotherapy	1	3.3
Selective internal radiation therapy (SIRT)	1	3.3

GEMCIS, gemcitabine plus cisplatin; CriPec^®^, docetaxel-entrapped core-crosslinked polymeric micelles

^a^One patient with liver and suspected positive locoregional lymph nodes

All patients received at least one cycle of the study treatment and received a median of 7.5 cycles (IQR 4.0–11.0). Median treatment duration, defined as the time from the first day of cycle one to the 14th day of the last cycle, was 3.8 months (IQR 2.2–5.8). Median treatment duration in stages one and two was 2.7 (2.2–5.4) and 3.9 (IQR 2.3–5.8) months, respectively.

In stage one, two patients experienced a treatment-related SAE, including one patient during the first six weeks of treatment. Partial response and stable disease were seen in one and five patients in stage one, respectively. In stage one, five of ten patients (50%) treated with FOLFIRINOX required a dose reduction within the first 6 weeks of treatment due to toxicities leading to initiation of stage two with a standard dose reduction and an adaptation of the treatment schedule for the remaining treatment cycles in stage one. Eight out of ten patients (80%) had at least one dose reduction of one or more treatment agents in stage 1 (oxaliplatin five patients (50%), irinotecan eight patients (80%), fluorouracil seven patients (70%)). The total number of dose reductions of oxaliplatin, irinotecan and fluorouracil bolus and fluorouracil continuous infusion in stage one was 8 (25.8%), 11 (35.5%), 7 (22.6%) and 5 (16.1%), respectively. All patients in stage one had at least one treatment interruption and the median number of treatment interruptions was 1.5 (IQR 1.0–2.0) resulting in a median of 14.5 days (IQR 14.0–19.8) of treatment delay. Median relative dose intensity, defined as the ratio of received cumulative dose and cumulative dose specified in the protocol, of oxaliplatin, irinotecan, fluorouracil bolus and fluorouracil continuous infusion in stage one was 95.0% (IQR 87.8–100), 87.2% (IQR 81.0–92.7), 51.3% (IQR 27.8–97.9) and 100% (IQR 90.4–100), respectively. Most common, clinically relevant grade 3–4 adverse events in stage one were neutropaenia (70%), diarrhoea (30%), mucositis (20%) and peripheral sensory neuropathy (20%) (Table 2). Four patients (40%) had a SAE not related to the study treatment in stage one, including one death due to abdominal sepsis.

**Table 2:** Treatment-related adverse events.

	Stage 1 (*N* = 10)			Stage 2 (*N* = 20)		
	Grade 1–2	Grade 3	Grade 4	Grade 1–2	Grade 3	Grade 4
Haematological						
Neutropenia	6 (60%)	7 (70%)	0	10 (50%)	6 (30%)	2 (10%)
Febrile neutropenia	0	1 (10%)	0	0	0	0
Anaemia	10 (100%)	1 (10%)	0	17 (85%)	3 (15%)	0
Thrombocytopenia	7 (70%)	0	0	9 (45%)	2 (10%)	1 (5%)
Non-haematological						
Fatigue	7 (70%)	0	0	18 (90%)	1 (5%)	0
Diarrhoea	6 (60%)	3 (30%)	0	13 (65%)	1 (5%)	0
Nausea	5 (50%)	0	0	18 (90%)	1 (5%)	0
Vomiting	4 (40%)	0	0	11 (55%)	1 (5%)	0
Anorexia	7 (70%)	1 (10%)	0	15 (75%)	0	0
Mucositis	7 (70%)	2 (20%)	0	8 (40%)	0	0
Peripheral sensory neuropathy	10 (100%)	2 (20%)	0	17 (85%)	0	0
Dizziness	2 (20%)	0	0	2 (10%)	0	0
Alopecia	3 (30%)	0	0	4 (20%)	0	0
Dyspnoea	3 (30%)	0	0	5 (25%)	0	0
Laryngitis	0	1 (10%)	0	0	0	0
Biochemical event^**a**^						
Increased alanine amino-transferase level	7 (70%)	3 (30%)	0	9 (45%)	0	0
Increased alkaline phosphatase level	3 (30%)	8 (80%)	1 (10%)	2 (10%)	1 (5%)	0
Increased ɣ-glutamyl-transferase level	1 (10%)	6 (60%)	2 (20%)	4 (20%)	3 (15%)	1 (5%)
Hypocalcemia	0	0	0	2 (10%)	1 (5%)	0
Hypomagnesaemia	0	0	0	2 (10%)	2 (10%)	0

Data are presented as the number of patients (*N*) (%). Grade 1 and 2 adverse events reported in at least 10% of patients and all grade 3 and 4 events are presented in this table. No treatment-related grade 5 events were occurred. Adverse events that occurred multiple times in an individual were counted only once.

^a^It was unclear whether these liver function abnormalities were related to the study treatment.

In stage two, 17 patients (85%) had at least one dose reduction, mostly for oxaliplatin (17 patients (85%)), followed by irinotecan (six patients (30%)) and fluorouracil (four patients (20%)). The total number of dose reductions of oxaliplatin, irinotecan and fluorouracil in stage two was 20 (66.7%), 6 (20.0%) and 4 (13.3%), respectively. Fifteen patients (75%) had a median number of 1.0 (IQR 0.75–2.0) treatment interruption and a median of 8.5 days (IQR 3.0–18.2) delay before receiving the next treatment cycle. Median relative dose intensity of oxaliplatin, irinotecan and fluorouracil in stage two was 81.2% (IQR 75.8%–93.1%), 100% (98.5%–100%) and 100% (IQR 100%–100%), respectively. Grade 3–4 adverse events were less common in stage two than in stage one and included neutropaenia (40%), thrombocytopenia (15%) and anaemia (15%). Three patients (15%) in stage two had a treatment-related SAE and nine patients (45%) had a SAE not related to the study treatment, including one death after euthanasia.

**Fig. 1:**
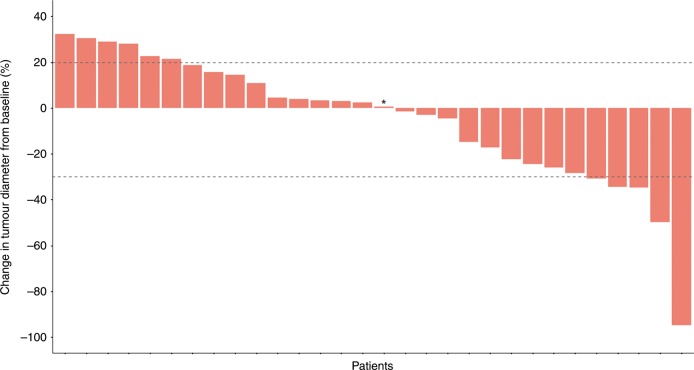
Change in tumour volume of target lesions. *, The tumour diameter of this patient did not change over time. The dashed lines represent 20% increase or 30% decrease in tumour diameter.

**Fig. 2:**
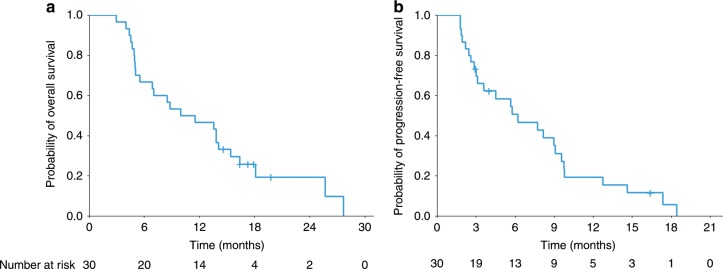
Kaplan–Meier curves of overall survival and progression-free survival. (a) overall survival (b) progression-free survival.

At the end of the follow-up period, all patients had discontinued the planned treatment because of disease progression (25 patients, 83.3%), unacceptable adverse events (one patient, 3.3%), or completion of planned treatment (four patients, 13.3%). Three patients (10%) had an ORR (three partial responses), 17 patients (57%) had stable disease and ten patients (33%) had progressive disease according to RECIST1.1. Fourteen patients (46.7%) had disease control of at least 4 months. Best change in tumour volume of the target lesions is presented in Fig. 1. After a median follow-up of 19.8 months (IQR 14.6–not reached), median PFS and OS in all enrolled patients were 6.2 (95% CI 3.0–9.1) and 10.7 (95% CI 5.5–15.4) months, respectively (Fig. 2). The OS at 6 and 12 months was 66.7% and 46.7%, respectively. Median OS for sequential GEMCIS followed by FOLFIRINOX was 18.5 months (95% CI 13.5–21.4).

In the univariate analysis of PFS and OS, patients with locally advanced disease had longer PFS and OS compared to those with metastatic disease. Patients with intrahepatic cholangiocarcinoma and gallbladder carcinoma had shorter PFS compared to those with perihilar cholangiocarcinoma (Supplementary Table 1).

Nine patients (30%) received third-line treatment. In two patients who had disease progression after experiencing a clinical benefit from study treatment (partial response and stable disease), FOLFIRINOX was reintroduced, achieving stable disease as best response on four and ten cycles, respectively.

## Discussion

In this phase 2 trial in patients with advanced BTC previously treated with GEMCIS, FOLFIRINOX was essentially safe (taking into consideration that the modified FOLFIRINOX was administered) as salvage treatment and resulted in an ORR of 10%. Fourteen patients (46.7%) had disease control of at least 4 months. The observed PFS and OS in our cohort of patients were 6.2 and 10.7 months, respectively.

Our findings showed the longest PFS and one of the best OS outcomes in the second-line setting compared to previous phase 2 trial of second-line chemotherapy and/or targeted therapy in advanced BTC previously treated with gemcitabine-based chemotherapy.^9,21–23^ There are no prospective studies using second-line FOLFIRINOX in advanced BTC. Two retrospective studies have combined patients treated with FOLFIRINOX in the first-line as well as in the second-line and demonstrated a median PFS and OS of 2.1–5.0 and 5.4–14.5 months, respectively.^15,24^ In the first-line, three retrospective studies evaluated FOLFIRINOX in advanced BTC and found a median PFS of 6.0–9.0 months, median OS of 10.0–15.0 months and an ORR of 36%–50%.^16,17,25^ The difference in ORR between these retrospective studies and our study could be partly explained by the fact that more responses are observed in first-line than in the second-line. Moreover, previous studies have shown that it is difficult to adequately asses the objective response in advanced BTC because the tumours are not well demarcated and show infiltration of the surrounding tissue.^26^ A pooled analysis of clinical trials showed a poor correlation between ORR and OS.^26^ Interestingly, another phase 2 trial studied a combination comparable to FOLFIRINOX but with S-1 instead of fluorouracil as first- or second-line treatment in advanced BTC and found similar median PFS and OS.^25^ It should, however, be noted that most studies had small sample sizes and were carried out in the first-line setting. The ongoing phase 2/3 AMEBICA trial is exploring the efficacy and safety of first-line FOLFIRINOX versus GEMCIS in patients with advanced BTC.^27^

Recently the results of the ABC-06 phase 3 clinical trial were presented at the American Society of Clinical Oncology (ASCO) annual meeting.^4^ This trial randomised 162 patients previously treated with GEMCIS between second-line modified FOLFOX versus ASC. Patients treated with modified FOLFOX had a median OS of 6.2 months compared to 5.3 months in the ASC-only arm. Six- and 12-months OS rates were significantly longer in the modified FOLFOX arm (50.6% and 25.9%) than in the ASC arm (35.5% and 11.4%). The authors recommended modified FOLFOX as the standard second-line treatment. Median OS and PFS of modified FOLFIRINOX in our study were longer compared to those in the ABC-06 trial.

Some targeted therapy agents showed promising results in patients with advanced BTC previously treated in the first-line setting.^28–31^ Ivosidenib, an isocitrate dehydrogenase-1 (IDH1) inhibitor, was well tolerated and showed a median PFS of 3.8 months and a median OS of 13.8 months in 73 patients with IDH1-mutant cholangiocarcinoma.^28^ An ongoing phase 3 trial (NCT02989857) is evaluating the efficacy of ivosidenib versus placebo in cholangiocarcinoma with IDH1 mutation. Two phase 2 trials studied the efficacy of fibroblast growth factor receptor (FGFR) inhibitors in intrahepatic cholangiocarcinoma with FGFR2 aberrations and found a median PFS of 5.8 months^29^ and an ORR of 14.8–21.0%.^29,30^ Dabrafenib, a BRAF inhibitor, combined with trametinib, a MEK inhibitor, showed an ORR of 41%, a median PFS of 7.2 months and a median OS of 11.3 months in patients with BRAF V600E-mutated BTC previously treated in the first line.^31^

FOLFIRINOX was well tolerated after initiation of standard dose reduction in stage two. Only one patient discontinued treatment due to toxicity (fatigue) after receiving one cycle of modified FOLFIRINOX. The most common grade 3–4 haematologic adverse event in patients treated with standard dose of FOLFIRINOX was neutropaenia (70% grade 3). However, this adverse event was less frequently observed after initiation of modified FOLFIRINOX in stage two (40%). Most common non-haematological grade 3–4 adverse events in stage one and two of our study were gastrointestinal toxicities, including diarrhoea, nausea, vomiting and mucositis.

To our knowledge, this is the first phase 2 clinical trial in advanced BTC using FOLFIRINOX and the first prospective study evaluating FOLFIRINOX as a salvage therapy. The percentages of each primary site of BTC enrolled in this study represent the disease distribution as seen in clinical practice. However, we are aware that the results of our study may be biased by selection given its non-randomised design.

In conclusion, our results suggest that a modified FOLFIRINOX schedule is safe and currently one of the most effective salvage treatments in patients with advanced BTC following treatment with GEMCIS. Considering the tolerability of 85 mg/m^2^ oxaliplatin, 400 mg/m^2^ leucovorin, 150 mg/m^2^ irinotecan and 2400 mg/m^2^ fluorouracil without a bolus, this treatment schedule should be considered in the second-line treatment of advanced BTC. A phase 3 trial could be initiated to compare modified FOLFIRINOX with modified FOLFOX.

## Supplementary information

Supplementary table 1

## Data Availability

Anonymous individual data and the study protocol could be requested from the corresponding author.
