# Supplementary material for: Efficacy and safety of FOLFIRINOX as salvage treatment in advanced biliary tract cancer: an open-label, single arm, phase 2 trial
Source: Br J Cancer. 2020 Jan 10;122(5):634–9. doi: 10.1038/s41416-019-0698-9 (PMC7054309; doi:10.1038/s41416-019-0698-9)
Supplement: Supplementary file 1 — Supplementary table 1 [file 41416_2019_698_MOESM1_ESM.docx]

## Supplementary Table 1: Univariate analyses

|  | **Overall survival** | | **Progression-free survival** | |
| --- | --- | --- | --- | --- |
|  | **Hazard ratio (95% CI)** | ***P-*value** | **Hazard ratio (95% CI)** | ***P-*value** |
| Age | 1.01 (0.97–1.05) | 0.681 | 1.01 (0.97–1.06) | 0.586 |
| Female sex | 0.74 (0.31–1.77) | 0.503 | 0.99 (0.44–2.21) | 0.972 |
| ECOG PS 1 (ref. ECOG PS 0) | 1.20 (0.49–2.91) | 0.696 | 1.53 (0.65–3.61) | 0.328 |
| Primary tumor site (ref. perihilar cholangiocarcinoma) |  |  |  |  |
| Distal cholangiocarcinoma | 1.94 (0.62–6.06) | 0.255 | 2.56 (0.85-7.71) | 0.094 |
| Intrahepatic cholangiocarcinoma | 2.38 (0.71–7.96) | 0.160 | 3.65 (1.09–12.22) | **0.036** |
| Gallbladder cancer | 3.08 (0.96–9.94) | 0.059 | 3.48 (1.09–11.14) | **0.036** |
| Locally advanced disease (ref. metastatic disease) | 0.29 (0.09–0.99) | **0.049** | 0.28 (0.09–0.84) | **0.024** |
| Distant metastases (ref. liver only metastases) | 1.25 (0.47–3.34) | 0.660 | 1.49 (0.53–4.20) | 0.448 |
| ≥ 2 metastatic sites (ref. 1 metastatic site) | 1.15 (0.47–2.85) | 0.762 | 1.03 (0.39–2.71) | 0.959 |
| Previous curative-intent surgery (ref. no surgery) | 2.31 (1.01–5.28) | **0.048** | 1.75 (0.77–3.97) | 0.180 |
| >6 cycles of GEMCIS (ref. ≤ 6 cycles) | 1.15 (0.48–2.71) | 0.757 | 1.05 (0.44–2.50) | 0.906 |
| Best objective response to GEMCIS (ref. partial plus stable disease) |  |  |  |  |
| Progressive disease | 0.73 (0.28–1.89) | 0.517 | 0.51 (020–1.30) | 0.157 |
| Unknown | 0.58 (0.13–2.54) | 0.471 | 0.51 (0.14–1.85) | 0.306 |
| Time between last dose of GEMCIS and start of FOLFIRINOX | 0.96 (0.89–1.02) | 0.202 | 0.94 (0.88–1.01) | 0.090 |
| CA 19-9 concentration > 667 U/ml (ref. ≤ median CA19-9 (≤ 667)) | 1.29 (0.57–2.95) | 0.539 | 0.76 (0.34-1.69) | 0.506 |

95% CI, 95% confidence interval; ref., reference.
